# PAFit: A Statistical Method for Measuring Preferential Attachment in Temporal Complex Networks

**DOI:** 10.1371/journal.pone.0137796

**Published:** 2015-09-17

**Authors:** Thong Pham, Paul Sheridan, Hidetoshi Shimodaira

**Affiliations:** 1 Division of Mathematical Science, Graduate School of Engineering Science, Osaka University, Osaka, Japan; 2 The Institute of Medical Science, The University of Tokyo, Tokyo, Japan; Tianjin University, CHINA

## Abstract

Preferential attachment is a stochastic process that has been proposed to explain certain topological features characteristic of complex networks from diverse domains. The systematic investigation of preferential attachment is an important area of research in network science, not only for the theoretical matter of verifying whether this hypothesized process is operative in real-world networks, but also for the practical insights that follow from knowledge of its functional form. Here we describe a maximum likelihood based estimation method for the measurement of preferential attachment in temporal complex networks. We call the method PAFit, and implement it in an R package of the same name. PAFit constitutes an advance over previous methods primarily because we based it on a nonparametric statistical framework that enables attachment kernel estimation free of any assumptions about its functional form. We show this results in PAFit outperforming the popular methods of Jeong and Newman in Monte Carlo simulations. What is more, we found that the application of PAFit to a publically available Flickr social network dataset yielded clear evidence for a deviation of the attachment kernel from the popularly assumed log-linear form. Independent of our main work, we provide a correction to a consequential error in Newman’s original method which had evidently gone unnoticed since its publication over a decade ago.

## Introduction

The *de facto* maxim of network science is ‘all systems are networks.’ For those who are well-acquainted with this network view of reality, recognizing the network nature of virtually any real-world system borders on reflexive. But network scientists are not interested in studying just any old networks. Network scientists habitually confine themselves to the study of complex networks, that is, large-scale networks with emergent topological features that are not found to occur in simple networks [1]. Complex network research turns more or less on the study of two related problems: first, the modelling of dynamical processes taking place on static complex networks in a manner that makes judicious use of known topological features [2], and second, the study of how topological features emerge in temporal complex networks and subsequently evolve over time [3]. It is the latter problem that concerns us in this paper. To be plain: we focus on understanding the extent to which a process know as preferential attachment (PA) explains the emergence of those heavy-tailed degree distributions, best exemplified by power-laws, that are commonly observed in temporal complex networks across nature, society, and technology [3].

It is reasonable to describe a wide variety of real-world systems as temporal (complex) networks. In formal terms, a temporal network represents an evolving system as a sequence of static (complex) networks. This sequence, which we denote by *G*_0_, *G*_1_, …, *G*_*T*_, is best envisioned as a progression of snapshots of a given system taken at discrete time-steps, *t* = 0, 1, …, *T*. The model at this level of generality is purely descriptive and serves only to frame our thinking about complex systems. Raising it up to the status of an explanatory model with predictive power requires the setting forth of laws governing node generation and the formation of node-to-node connections in the transition from *G*_*t*−1_ to *G*_*t*_. This, however, is a subject to which we must return after a brief digression on some important empirical findings.

By the late 1990s, temporal networks in diverse domains of learning were observed to enjoy some universal topological features [4]. The most publicized of these features at the time was the so-called scale-free property [5]. A network, it will be noted, is scale-free when its degree distribution follows a particular kind of heavy-tailed function called a power-law [6]. The purported universality of scale-free networks was soon, however, called into question when it was pointed out that distinguishing a power-law degree distribution from alternative heavy-tailed functional forms, such as the log-normal distribution, is a matter of considerable subtlety [5–8]. In light of this consideration, we will proceed on the supposition that the temporal networks in the world around us are commonly found to have heavy-tailed degree distributions with those following power-laws constituting an interesting special case.

What was interpreted as mounting empirical evidence for scale-free networks had everybody in network science scurrying to propose explanatory temporal network models that account for them in terms of domain independent processes. The first and most influential of these is known as the Barabási-Albert (BA) model [4]. It is essentially a toy model that generates scale-free networks by appealing to a simple growth law for node generation and a special case of PA to govern the formation of node-to-node connections.

Consider a generalization of the BA model that allows for networks with a wider range of heavy-tailed degree distributions [9]. Let *G*_*t*_ denote a static network at time-step *t* = 0, 1, …, *T*. Starting from a seed network *G*_0_, for each time-step 1 ≤ *t* < *T*, the network *G*_*t*−1_ is ‘grown’ by a new node *v*′ that is subsequently connected to an already existing node to form *G*_*t*_. Note that, in order to review the precise results about the degree distribution, here we only consider the case in which one node is added at each time-step. Later in the Materials and Methods section, we will describe a model that more closely reflects real-world networks, in which multiple new nodes can appeared at the same time. The PA rule then states that *v*′ connects to a node *v*_*k*_ of degree *k* in *G*_*t*−1_ with probability proportional to *A*_*k*_:

$$\begin{array}{c} Pr\left(v^{\prime }\;\;\text{connects}\;\text{to}\;v_{k}\right)\propto A_{k}. \end{array}$$ (1)

The function *A*_*k*_ is called the *attachment kernel*. Strictly speaking, PA is only said to occur when *A*_*k*_ is an increasing function on average. It has been shown that the functional form of the attachment kernel can have dramatic effects on temporal network topology. Let us consider the log-linear model *A*_*k*_ = *k* ^*α*^ with *attachment exponent* *α* > 0 as an important example. When *A*_*k*_ = *k* (*α* = 1) the resulting network is scale-free [9]. This is called *linear* PA and it is this form that it is assumed in the BA model. On the other hand, *sub-linear* PA (0 < *α* < 1) gives rise to a class of stretch-exponential degree distributions, while *super-linear* PA (*α* > 1) leads to a winner-take-all phenomena whereby a handful of nodes acquire all the connections [9]. The limiting case *A*_*k*_ = 1 for all *k* (*α* = 0) corresponds to a version of the classical Erdös-Rényi random network model [10]. In this case, the network is also not scale-free.

The point is that the Barabási-Albert family of models explain the emergence of a robust class of heavy-tailed degree distributions in temporal networks in terms of two generic processes: growth and PA. It is, moreover, plausible that these processes operate in many real-world temporal networks. In expanding social networks, for example, it is reasonable to presume that a person with many acquaintances already will tend to make new ones at a faster rate, than a person with few. Or, in citation networks of research papers, to take another example, a paper with many citations may acquire new citations more readily, than a comparatively lesser known paper. The same argument is easily specialized to other sorts of temporal networks by reasoning along similar lines.

Let us suppose for the sake of argument that heavy-tailed degree distributions of this kind prevail in growing temporal networks in the real world. This would not prove that PA was responsible for their generation, because it is always possible that a different process was at work. Rather, the presence of PA in the generative process must be confirmed by observation. In practice, this amounts to estimating the attachment kernel, *A*_*k*_, from observed data.

The problem of estimating *A*_*k*_ from observed data is one of great importance to the working network scientist. For one thing, there are practical applications in link prediction algorithms [11], and more generally, it gives us valuable insights into the global characteristics of networks [9]. But the core questions surrounding the PA process are these: does PA actually exist in real-world temporal networks; or, in other words, is *A*_*k*_ really increasing in *k*? If so, then what elementary functional form, if any, does it take? Is it the widely accepted log-linear model *A*_*k*_ = *k* ^*α*^, or something else? In order to investigate these questions scientifically, a rigorous statistical method to estimate the functional form of *A*_*k*_ with confidence bounds is necessary. However, to the best of our knowledge, no such method has yet been advanced in the literature.

Nevertheless, detecting the hand of PA in temporal networks has attracted the attention of many researchers, and a number of estimation methods for *A*_*k*_ have been proposed [12–17]. Table 1 shows a summary of the existing methods. The primary drawback of most of these methods is that they explicitly assume the log-linear form *A*_*k*_ = *k* ^*α*^ and focus only on estimating the attachment exponent *α* [14–17]. Massen et al. [14] used a fixed point iterative algorithm for the purpose, Sheridan et al. [15] a Markov chain Monte Carlo method, Gomez et al. [16] a maximum likelihood (ML) approach to estimate the value of *α*. Lastly, Kunegis et al. [17] estimated *α* by fitting a function that relates the number of new connections a node obtains in the future with its current degree.

**Table 1:** Summary of the existing attachment kernel estimation methods.

Method	Form of *A*_*k*_	Estimation method
Newman [12]	Nonparametric	Weighted sum of multiple histograms
Jeong et al. [13]	Nonparametric	Single histogram
Massen et al. [14]	*A*_*k*_ = *k* ^*α*^	Iterative fixed-point algorithm
Sheridan et al. [15]	*A*_*k*_ = *k* ^*α*^	Markov chain Monte Carlo
Gomez et al. [16]	*A*_*k*_ = *k* ^*α*^	ML by grid search
Kunegis et al. [17]	*A*_*k*_ = *k* ^*α*^	Regression

Summary of the existing methods for estimating the attachment kernel *A*_*k*_. Nonparametric methods are methods that do not assume a functional form for *A*_*k*_.

The remaining methods [12, 13] do not assume any functional form for *A*_*k*_. As invaluable as these methods are, they are ad-hoc methods that are not without their problems. Newman’s method [12] is among the first methods proposed to estimate *A*_*k*_. In real-world examples, Newman’s method appears to be able to estimate *A*_*k*_ well for small *k*, but systematically underestimates *A*_*k*_ when *k* is large [18, 19]. This is thought to be an artifact of the method [19]. We will explain the reason for this artifact in detail below. On the other hand, while Jeong’s method [13] is the most widely-used method in practice [19–31], as we will explain in the Materials and Methods section, it suffers from a kind of bias-variance trade-off.

Our contributions in this paper are twofold.

First and foremost, we propose a nonparametric estimation method called PAFit that employs the ML approach of [32] to fit the functional form of the attachment kernel. PAFit is nonparametric in the sense that it does not make any assumptions on the functional form of *A*_*k*_. The method works instead by estimating the value of *A*_*k*_ separately for each *k*. The algorithm underlying PAFit is the Minorize-Maximization [33, 34] (MM) algorithm. We prove that our algorithm converges to a global maximizer of the log-likelihood function and provide a fast approximation to the confidence intervals of the estimated attachment kernel ${\hat{A}}_{k}$. This last step is essential for the analysis of large datasets. What is more, we present the results of extensive Monte Carlo simulations that demonstrates how PAFit outperforms the state-of-the-art methods, namely Jeong’s method and Newman’s method, at the task of estimating *A*_*k*_ for a variety of different functional forms. Lastly, we used PAFit to analyze a publicly available Flickr social network dataset [35], and found that the estimated attachment kernel differed considerably from the functional form *A*_*k*_ = *k* ^*α*^. This result immediately suggests that it is important to look beyond the classical log-linear form when modelling the attachment kernel.

A second contribution of ours that should not be overlooked is an explanation of the artifact observed in the Newman’s method. In particular, we show that this artifact can be completely eliminated by a simple mathematical correction. This is important because researchers have been using Newman’s method to estimate *A*_*k*_ in real-world networks for the better part of 15 years. It will be noted that while PAFit outperformed the corrected version of Newman’s method in the simulated examples, the corrected version was found to work better than uncorrected version.

In summary, PAFit is a statistically sound method for estimation of the attachment kernel in temporal complex networks. We have demonstrated its potential in this paper to uncover interesting findings about real-world complex networks. This method is implemented in the R package PAFit [36].

Before entering into the details of PAFit in the next sections, we note some recent developments in the field of complex networks. In this paper, we estimate the growth mechanism, in particular the PA mechanism, of a network given its growth process, that is, the observed data are the final network and all previous networks in the growth process. There is another line of research that considers a somewhat opposite problem: the underlying network is unknown and the task is to estimate it. This line of research includes, for example, estimating the unknown gene regulatory network from observed omics data [37, 38]. Complex networks have also found applications in many other areas, including economics [39], image analysis [40] and epidemiology [2, 3, 41, 42]. Beside the scale-free property, which only concerns the degree distribution of a network, other topological features which concern higher order of information have also been studied extensively in the literature [43–46].

## Materials and Methods

In this section, we begin by introducing the temporal network model underlying the PAFit method. We follow that with an illustrative example to demonstrate the workings of PAFit in comparison with previous methods. From there we explain the mathematical details of PAFit and call attention to its important properties. The similarities between PAFit and previous methods are discussed. It is there that the correction to Newman’s method is outlined. Next, fast calculations for the confidence intervals of the estimated values are explained. We then proceed to discuss binning and regularization, which are two important techniques for stabilizing the PAFit estimation. Finally, we mention how to estimate the attachment exponent *α* of the log-linear model *A*_*k*_ = *k* ^*α*^ from ${\hat{A}}_{k}$.

### The general temporal model

The statistical estimation method we present in this paper is tailored for the following temporal model for directed networks. Note, however, that our method can be easily adapted to work for undirected networks. The model is a simplified version of a model that we introduced in a previous publication [32]. Starting from a seed network at time-step *t*_0_ = 0, we grow the network by adding *n*(*t*) nodes and *m*(*t*) edges at every time-step *t*, for *t* = 1, …, *T*. Note that our method allows *m*(*t*) to be consisted of both new edges that emanate from the *n*(*t*) new nodes and emergent edges between existing nodes. This is important since a large number of real-world networks have emergent edges between existing nodes. At time-step *t*, the probability that an existing node *v* with in-degree *k* acquires a new edge is

$$\begin{array}{c} Pr\left(v\;\;\text{acquires}\;\text{a}\;\text{new}\;\text{edge}\right)\propto A_{k}, \end{array}$$ (2)

where *A*_*k*_ is the value of the attachment kernel at degree *k*. We call this model the general temporal (GT) model. The GT model includes a number of important network models as special cases. When *A*_*k*_ = *k* ^*α*^, the GT model corresponds to Price’s model [47, 48] or Barabási-Albert (BA) model [4] in the undirected case. Furthermore, when *A*_*k*_ = 1 for all *k*, then it reduces to the classical Erdös-Rényi random network model [10].

Here we note a quick remark on an assumption about the joint probability distribution of *n*(*t*) and *m*(*t*). We will return to this assumption later when we discuss the MLE for *A*_*k*_. Let ***θ***(*t*) denote the parameter vector that governs the joint distribution of *n*(*t*) and *m*(*t*). We assume that ***θ***(*t*) does not involve *A*_*k*_. This very mild assumption allows broad and realistic models for *m*(*t*) and *n*(*t*). For example, *m*(*t*) and *n*(*t*) can be random variables whose means and variances depend on *G*_*t*−1_.

A final remark is that, although we do not incorporate deletions of nodes and edges into the formal specification of the GT model, this is purely for simplicity. The PAFit method is able to estimate *A*_*k*_ even when there are deletions of nodes and edges, as long as the deletion mechanism is independent of the addition mechanism and the parameters govern the deletion mechanism do not involve *A*_*k*_, which are reasonable assumptions. The current R implementation of PAFit [36] can be easily adapted to work in these cases.

### An illustrative example

In this section we present a simulated example of a GT model network. The purpose being to demonstrate the workings of our proposed method, PAFit. The network generative process follows the GT model with *A*_*k*_ = 3(log(max(*k*, 1)))^2^ + 1. Starting from a seed network with 20 nodes, at each time-step, *m*(*t*) = 5 new edges and *n*(*t*) = 1 new node were added, until a total of *T* = 2000 nodes is reached. We measure how well a method performs by using the average relative error between the true *A*_*k*_ and the estimated ${\hat{A}}_{k}$, defined as $e_{A}=\frac{1}{K}\sum_{k=1}^{K}\frac{{\left(A_{k}-{\hat{A}}_{k}\right)}^{2}}{A_{k}^{2}}$ where *K* is the maximum degree that appears in the growth process.

**Fig 1:**
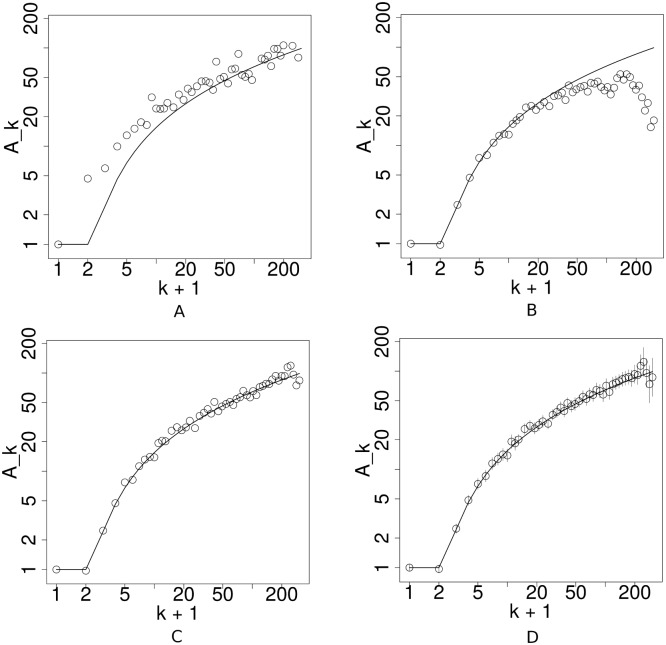
Estimation of the attachment kernel when the true model is *A*_*k*_ = 3(log(max(*k*, 1)))^2^ + 1. A: Jeong’s method. B: Newman’s method. C: Corrected Newman’s method. D: PAFit. The solid line depicts the true model. The plots are on a log-log scale. The gray vertical lines are the estimated confidence intervals of the estimated values by PAFit. Confidence intervals are not available in the remaining methods.

We first apply Jeong’s method. Here we choose the time window between when the 1500-th and 2000-th nodes are added. After a suitable normalization, the histogram of the degrees of nodes to which new edges appeared in this window connect will give us ${\hat{A}}_{k}$. The average relative error *e*_*A*_ for Jeong’s method is 0.337, which is the highest among four methods considered in this example. From Fig 1A, one can see that Jeong’s method did capture the shape of *A*_*k*_, but the estimated function was sparse and fluctuated considerably. These are inherent drawbacks of the method that follow from using only a small time window to estimate *A*_*k*_.

Second, we apply Newman’s method. His method can be interpreted as estimating the attachment kernel by a weighted sum of multiple histograms created at multiple time-steps. In Fig 1B, ${\hat{A}}_{k}$ follows the true function very closely, but the value of *A*_*k*_ starts to fall off when the degree *k* becomes large. This phenomenon can also be seen in other papers [18, 19], and is thought to be an artifact of the method [19]. The value of *e*_*A*_ for Newman’s method is 0.168, which is the second highest error among four methods used in this example. With a simple correction, we can eliminate this artifact. As can be seen from Fig 1C, the corrected Newman’s method estimated the attachment kernel well the entire range of *k*. The value of *e*_*A*_ for this corrected version is 0.016, which is much smaller than those of Jeong’s method and the original method of Newman.

Unlike all the other methods, PAFit estimates the attachment kernel by maximum likelihood estimation (MLE), and can give quantifications of the uncertainties in the value of ${\hat{A}}_{k}$ in the form of confidence intervals. In Fig 1D, the estimated attachment kernel follows the true function comparatively well, even in the high degree region. The average relative error *e*_*A*_ of PAFit is 0.015, which is the smallest error among four method considered here.

### Maximum likelihood estimation

Here we derive the MLE for the GT model as described in the previous section. Recall that *K* is the maximum degree that appears in the growth process. Let **A** = [*A*_0_, *A*_1_, …, *A*_*K*_] be the parameter vector we want to estimate. The likelihood of the data at time-step *t* ≥ 1 is the product of *P*(*m*(*t*), *n*(*t*)∣*G*_*t*−1_, ***θ***(*t*)) and *P*(*G*_*t*_∣*G*_*t*−1_, *m*(*t*), *n*(*t*), **A**). The likelihood of the data at the starting time *t* = 0 is the product of *P*(*m*(0), *n*(0)∣***θ***(0)) and *P*(*G*_0_∣*m*(0), *n*(0), θ_*_), with θ_*_ the parameter vector governing the distribution of *G*_0_. Thus the log-likelihood function of the dataset is

$$\begin{array}{ccc} l(A) & = & \sum_{t=1}^{T}\log P\left(G_{t}|G_{t-1},m\left(t\right),n\left(t\right),A\right)+\sum_{t=1}^{T}\log P\left(m\left(t\right),n\left(t\right)|G_{t-1},\theta \left(t\right)\right)+\\ & & \log P\left(m\left(0\right),n\left(0\right)|\theta \left(0\right)\right)+\log P\left(G_{0}|m\left(0\right),n\left(0\right),\theta_{*}\right). \end{array}$$ (3)

As previously mentioned, we assume that ***θ***(*t*), the parameter vector governing the joint distribution of *m*(*t*) and *n*(*t*), does not involve **A**. We also assume that θ_*_ does not involve **A**. When deriving the MLE of **A**, these two assumptions allow us to ignore all but the first term in the right hand side of Eq (3). At the onset of time-step *t*, denote *m*_*k*_(*t*) and *n*_*k*_(*t*) as the number of new edges that connect to a node with degree *k* and the number of existing nodes with degree *k*, respectively. The term *P*(*G*_*t*_∣*G*_*t*−1_, *m*(*t*), *n*(*t*), **A**) corresponds to a multinomial distribution. This follows from the observation that given *m*(*t*), the quantities *m*_0_(*t*), *m*_1_(*t*), …, *m*_*K*_(*t*) follow a multinomial distribution with parameters *p*_0_(*t*), *p*_1_(*t*), …, *p*_*K*_(*t*), where *p*_*k*_(*t*), the probability that a newly added edge at time *t* connects to a node with degree *k*, is

$$\begin{array}{c} p_{k}\left(t\right)=\frac{n_{k}\left(t\right)A_{k}}{\sum_{j=0}^{K}n_{j}\left(t\right)A_{j}}. \end{array}$$ (4)

After dropping constant terms, we can write down the log-likelihood function in detail:

$$\begin{array}{c} l\left(A\right)=\sum_{t=1}^{T}\sum_{k=0}^{K}m_{k}\left(t\right)\log A_{k}-\sum_{t=1}^{T}m\left(t\right)\log(\sum_{j=0}^{K}n_{j}\left(t\right)A_{j}). \end{array}$$ (5)

Note that the *A*_*k*_ are only identifiable up to a multiplicative constant, as can be seen from Eq (4). We can enforce uniqueness by fixing *A*_*k*_ = 1 for some *k* that satisfies $\sum_{t=1}^{T}m_{k}(t)>0$. In practice, we can often set, for example, *A*_1_ = 1.

We find the MLE by solving the following likelihood equation.

$$\begin{array}{c} \partial l\left(A\right)/\partial A=0⟺A_{k}=\frac{\sum_{t=1}^{T}m_{k}\left(t\right)}{\sum_{t=1}^{T}\frac{m\left(t\right)n_{k}\left(t\right)}{\sum_{j=0}^{K}n_{j}\left(t\right)A_{j}}}\;k=1,\ldots ,K. \end{array}$$ (6)

But before that we have the following observation.

**Proposition 1** *Any* **A**′ *that satisfies* ∂*l*(**A**)/∂**A** = 0 *is a global maximizer of* *l*(**A**).

The implication of this proposition is that even though the log-likelihood function *l*(**A**) might be not concave, any local maximizer is guaranteed to be a global maximizer.

*Proof.* We consider the one-to-one re-parametrization ***β*** = ***β***(**A**) = [log*A*_0_, …, log*A*_*K*_]^*T*^. From Eq (5) and the inverse relation *A*_*k*_ = exp*β*_*k*_, the log-likelihood function under this new parametrization is

$$\begin{array}{c} l_{\beta }\left(\beta \right)=\sum_{t=1}^{T}\sum_{k=0}^{K}m_{k}\left(t\right)\beta_{k}-\sum_{t=1}^{T}m\left(t\right)\log(\sum_{j=0}^{K}n_{j}\left(t\right)\exp\beta_{j}). \end{array}$$

The second derivative of the log-sum-exponential term $\text{log}\left(\sum_{j=0}^{K}n_{j}(t)\text{exp}\beta_{j}\right)$ can be shown to be non-negative semi-definite (a similar calculation can be found on page 74 of [49]). Therefore the second derivative Δ^2^ *l*_***β***_(***β***) of *l*_***β***_(***β***) is non-positive semi-definite. Thus *l*_***β***_(***β***) is concave. Now the likelihood equation of the re-parametrized log-likelihood function *l*_***β***_ is

$$\begin{array}{c} \partial l_{\beta }/\partial \beta =0⟺\exp\beta_{k}=\frac{\sum_{t=1}^{T}m_{k}\left(t\right)}{\sum_{t=1}^{T}\frac{m\left(t\right)n_{k}\left(t\right)}{\sum_{j=0}^{K}n_{j}\left(t\right)\exp\beta_{j}}}\;k=1,\ldots ,K. \end{array}$$ (7)

One can see that any **A**′ satisfying Eq (6) will give a reparametrization ***β***′ that satisfies Eq (7). This ***β***′ is a global maximizer since *l*_***β***_(***β***) is concave. Thus the **A**′ is also a global maximizer.

Turning now to how to solve for *A*_*k*_, since *A*_*k*_ appears in both sides of Eq (6), an explicit solution for **A** is difficult to obtain. Define ${\text{A}}^{\left(i\right)}=\left[A_{1}^{\left(i\right)},\ldots ,A_{K}^{\left(i\right)}\right]$ as the estimated parameter vector at iteration *i*. Starting from some initial value **A** ^(0)^, at iteration *i* ≥ 1 we can iteratively update $A_{k}^{\left(i\right)}$ using Eq (6):

$$\begin{array}{c} A_{k}^{\left(i\right)}=\frac{\sum_{t=1}^{T}m_{k}\left(t\right)}{\sum_{t=1}^{T}\frac{m\left(t\right)n_{k}\left(t\right)}{\sum_{j=0}^{K}n_{j}\left(t\right)A_{j}^{\left(i-1\right)}}}\;k=1,\ldots ,K. \end{array}$$ (8)

We repeat Eq (8) until the following convergence condition is met.

$$\begin{array}{c} \frac{\left|l\left(A^{\left(i\right)}\right)-l\left(A^{\left(i-1\right)}\right)\right|}{|l\left(A^{\left(i-1\right)}\right)|+1}\leq \epsilon . \end{array}$$ (9)

This iterative algorithm turns out to be an instance of Minorize-Maximization (MM) [33, 34] algorithms, which are used in maximization problems. In MM algorithms, instead of maximizing a complicated objective function, at each iteration we find and maximize a minorize function of the objective function. A minorize function is a function that is equal to the objective function at the current point, and is the lower bound of the objective function at all other points. The minorize function is often chosen so that we can maximize it easier than the original objective function. For our problem, we have the following proposition:

**Proposition 2** *Using the aforementioned algorithm, the log-likelihood function is guaranteed to increase with number of iterations. In particular,*

$$l\left(A^{\left(i\right)}\right)\leq l\left(A^{\left(i+1\right)}\right).$$

*Furthermore, the stopping point* **A** ^(*M*)^ *which satisfies* **A** ^(*M*)^ = **A** ^(*M*−1)^ *is a global maximizer of* *l*(**A**).

*Proof.* We find a minorize function *Q*_*i*_(**A**), which is a function that satisfies *l*(**A**) ≥ *Q*_*i*_(**A**) for all **A**, and *l*(**A** ^(*i*)^) = *Q*_*i*_(**A** ^(*i*)^). Define

$$\begin{array}{ccc} Q_{i}\left(A\right) & = & \sum_{t=1}^{T}\sum_{k=0}^{K}m_{k}\left(t\right)\log A_{k}-\sum_{t=1}^{T}m\left(t\right)\log(\sum_{j=0}^{K}n_{j}\left(t\right)A_{j}^{\left(i\right)})-\\ & & \sum_{t=1}^{T}m\left(t\right)\frac{\sum_{j=0}^{K}n_{j}\left(t\right)A_{j}}{\sum_{j=0}^{K}n_{j}\left(t\right)A_{j}^{\left(i\right)}}+\sum_{t=1}^{T}m\left(t\right). \end{array}$$ (10)

Applying the inequality −log*x* ≥ −log*y* − *x*/*y* + 1, ∀*x*, *y* > 0 with $x=\sum_{j=0}^{K}n_{j}(t)A_{j}$ and $y=\sum_{j=0}^{K}n_{j}(t)A_{j}^{\left(i\right)}$, one can verify that *l*(**A**) ≥ *Q*_*i*_(**A**). By substitution, one can check that *l*(**A** ^(*i*)^) = *Q*_*i*_(**A** ^(*i*)^). It is also easy to verify that the **A** ^(*i*+1)^ computed by Eq (8) is the solution of ∂*Q*_*i*_/∂**A** = 0, and thus satisfies **A** ^(*i*+1)^ = argmax *Q*_*i*_(**A**). Therefore,

$$l\left(A^{\left(i\right)}\right)=Q_{i}\left(A^{\left(i\right)}\right)\leq Q_{i}\left(A^{\left(i+1\right)}\right)\leq l\left(A^{\left(i+1\right)}\right).$$

Suppose that the algorithm stops at iteration *M* such that **A** ^(*M*)^ = **A** ^(*M*−1)^. From Eqs (8) and (6), we can see that **A** ^(*M*)^ satisfies Eq (6). Combining this with Proposition 1, this means that **A** ^(*M*)^ is a global maximizer *l*(**A**).

It is instructive to look at Eq (8). If we only have one time-step *t*, then the MLE solution is *A*_*k*_ = *m*_*k*_(*t*)/*n*_*k*_(*t*), which is an intuitive formula. When there are multiple time-steps, things become less obvious. In this case, the MLE solution for *A*_*k*_ sums up all the *m*_*k*_(*t*) then divides this number by a weighted sum of the *n*_*k*_(*t*). Looking at the weights, we notice that a weight is larger when the number of new edges *m*(*t*) is large and the normalizing factor $\sum_{j=0}^{K}n_{j}(t)A_{j}$ is small. This can be interpreted as the MLE solution naturally emphasizing *n*_*k*_(*t*) of the time-step *t* that has a lot of information.

### Related work

In this section, we discuss in detail the two most important methods for estimating the attachment kernel: Jeong’s method and Newman’s method. We then provide a simple mathematical correction to the Newman’s method. Finally, we discuss how PAFit fits in the framework of these previous methods.

#### Jeong’s method

Jeong’s method [13] estimates *A*_*k*_ by building a histogram of the degrees of nodes to which new edges connect. They choose a time window *T*_0_ and call all nodes in the network at that time *T*_0_-nodes. Next they choose a time window *T*_1_ > *T*_0_ and a window length Δ*T* ≪ *T*_1_, then call all nodes added in the interval [*T*_1_, *T*_1_ + Δ*T*] the *T*_1_-nodes. For a degree *k*, they record the number of times a degree *k* *T*_0_-node is linked to by a *T*_1_-node, and then normalize this number by *n*_*k*_(*T*_0_) to give ${\hat{A}}_{k}$. No guidelines are offered for choosing *T*_0_, *T*_1_ and Δ*T*.

This method very clearly suffers a type of bias-variance trade-off. In order to negate the effect of fixing the degrees of *T*_0_-nodes at time *T*_0_, one should use a very small window time Δ*T*. But if the number of new edges in Δ*T* is small, then the estimation will be unstable. We can increase the number of new edges by increasing the length of Δ*T*, but this will inevitably introduce a bias into the estimation.

One natural way to overcome this drawback is to use multiple histograms calculated at multiple time-steps, but then the question arises as to how to combine those histograms into a single estimation of *A*_*k*_ while the normalizing factor at each time-step depends on **A** (and thus unknown). Newman’s method provides an answer to this question.

#### Newman’s method

Instead of using only one histogram at a single time window, Newman’s method can be interpreted as estimating *A*_*k*_ by a weighted sum of multiple histograms created at multiple time-steps. Let *N*(*t*) denote the number of nodes in the network at the onset of time-step *t*. Consider creating a histogram at time-step *t*. Denote *A*_*k*_(*t*) as the value of *A*_*k*_ at time *t*. Newman’s argument, written in the notation used here, is that *A*_*k*_(*t*) can be estimated as

$$\begin{array}{c} A_{k}\left(t\right)=\frac{m_{k}\left(t\right)N\left(t\right)}{n_{k}\left(t\right)m\left(t\right)} \end{array}$$ (11)

if *n*_*k*_(*t*) ≠ 0 or *A*_*k*_(*t*) = 0 if *n*_*k*_(*t*) = 0, using the following approximations [12].

$$\begin{array}{c} \frac{m_{k}\left(t\right)}{m(t)}\approx p_{k}\left(t\right)\approx A_{k}\left(t\right)\frac{n_{k}\left(t\right)}{N(t)}. \end{array}$$ (12)

The final result of the procedure proposed by Newman [12] is equivalent to the formula

$$\begin{array}{c} A_{k}=\frac{1}{C}\sum_{t=1}^{T}w_{k}\left(t\right)A_{k}\left(t\right) \end{array}$$ (13)

with the weight *w*_*k*_(*t*) = *m*(*t*)1_*n*_*k*_(*t*) ≠ 0_, using the convention 0/0 = 0. Note that, in Newman’s argument, the normalized constant *C* in Eq (13) is a constant that does not depend on *k*. In real-world examples, this procedure seems to work well for small *k*, but the value of *A*_*k*_ starts to fall off when *k* is large [18, 19] (cf. Fig 1B). Although this phenomenon is often thought to be an artifact of the method [19], an explanation of its cause has yet to be provided.

#### The corrected Newman’s method

Surprisingly, we can completely eliminate the artifact in Newman’s original method by a applying a simple mathematical correction (cf. Fig 1C). Immediately from Eq (13), one can recognize that the summation is incorrectly normalized. The constant *C* should depend on *k*. The correct normalization is as follows:

$$\begin{array}{c} A_{k}=\frac{1}{\sum_{t}w_{k}\left(t\right)}\sum_{t=1}^{T}w_{k}\left(t\right)A_{k}\left(t\right). \end{array}$$ (14)

Newman’s original method (see Eq (13)) implicitly assumes that all the degrees already appear in the network from time *t* = 0. This is of course not generally true. In a typical network that grows from a small initial network, the small degrees will appear far sooner than the large degrees. In such cases, the sum ∑_*t*_ *m*(*t*)1_*n*_*k*_(*t*) ≠ 0_, which is a weighted sum of the number of time-steps that contain nodes with degree *k*, is larger for small *k* and smaller for large *k*. Forgetting to normalize each *A*_*k*_ in Eq (13) by ∑_*t*_ *m*(*t*)1_*n*_*k*_(*t*) ≠ 0_ will cause the waterfall artifact as observed in Fig 1B. In Eq (14), correctly normalizing each *A*_*k*_ by this number completely eliminates the artifact. We will prefer to Eq (14) as the *corrected Newman’s method*.

#### Relation of PAFit to previous methods

Using almost the same route as in deriving Newman’s method, one can deduce a similar equation to PAFit’s MLE Eq (6). Starting from Eq (12), *m*_*k*_(*t*) is equal to *A*_*k*_(*t*)*m*(*t*)*n*_*k*_(*t*)/*N*(*t*). Summing over all time-steps *t*, we see that $\sum_{t=1}^{T}m_{k}(t)$ is equal to $\sum_{t=1}^{T}A_{k}(t)m(t)n_{k}(t)/N(t)$. Assuming *A*_*k*_(*t*) = *A*_*k*_ for all *t*, we have

$$\begin{array}{c} A_{k}=\frac{\sum_{t=1}^{T}m_{k}\left(t\right)}{\sum_{t=1}^{T}\frac{m\left(t\right)n_{k}\left(t\right)}{N(t)}}. \end{array}$$ (15)

The only difference between Eqs (15) and (6) is the normalizing factor at time *t*: $\sum_{j=0}^{K}n_{j}(t)A_{j}$ versus *N*(*t*). This difference is the result of the approximation in Eq (12).

If the ratio $\left(\sum_{j=0}^{K}n_{j}(t)A_{j}\right)/N(t)$ is independent of *t*, then Eqs (15) and (6) are equivalent. Notice that $\sum_{j=0}^{K}n_{j}(t)A_{j}/N(t)$ is equal to $\sum_{j=0}^{K}A_{j}\left(n_{j}(t)/N(t)\right)$ where *n*_*j*_(*t*)/*N*(*t*) is the proportion of nodes with degree *j* at time *t*. Interestingly, this proportion has been shown to be independent of *t* when *t* is large in the case of asymptotically linear or sub-linear attachment kernels, that is, *A*_*k*_ ∼ *k* ^*α*^ with *α* ≤ 1 [9]. In such cases, Eqs (15) and (6) are asymptotically equivalent. The two equations are not asymptotically equivalent when *A*_*k*_ is super-linear, that is, *A*_*k*_ ∼ *k* ^*α*^ with *α* > 1, since in this case the proportion *n*_*j*_(*t*)/*N*(*t*) depends on *t* [9].

### Fast approximation of the confidence intervals

We provide a fast approximation for the confidence intervals of ${\hat{A}}_{k}$. Our approximation works quite well in practice. In many real-world datasets, our approximation can provide a huge gain in speed. In standard statistical theory, the confidence intervals of $\hat{\text{A}}$ can be calculated as the diagonal entries of the inverse of **D**, where **D** = −∂^2^ *l*(**A**)/∂**A**∂**A**. Define the following matrices **B**, **C**, **U** as:

$$\begin{array}{c} B=\text{diag}(\frac{\sum_{t=1}^{T}m_{1}\left(t\right)}{{\hat{A}}_{1}^{2}},\ldots ,\frac{\sum_{t=1}^{T}m_{K}\left(t\right)}{{\hat{A}}_{K}^{2}}),U=(\begin{array}{ccc} n_{1}\left(1\right) & \cdots & n_{1}\left(T\right)\\ \vdots & \ddots & \vdots \\ n_{K}\left(1\right) & \cdots & n_{K}\left(T\right) \end{array}), \end{array}$$

$$\begin{array}{c} C=\text{diag}(\frac{-m(1)}{\sum_{j=1}^{K}n_{j}\left(1\right){\hat{A}}_{j}},\ldots ,\frac{-m(T)}{\sum_{j=1}^{K}n_{j}\left(T\right){\hat{A}}_{j}}), \end{array}$$

then we have

$$\begin{array}{c} D=B+UCU^{T}. \end{array}$$ (16)

The form of Eq (16) makes it quite natural to calculate the inverse of **D** by the Woodbury formula [50]:

$$\begin{array}{c} D^{-1}={\left(B+UCU^{T}\right)}^{-1}=B^{-1}-B^{-1}U{\left(C^{-1}+U^{T}B^{-1}U\right)}^{-1}U^{T}B^{-1}. \end{array}$$ (17)

Note that **B** and **C** are both diagonal matrices. This makes inverting them trivial. A direct inversion of **D** will require *O*(*K* ^3^) operations, while using Eq (17) will require *O*(*KT* ^2^). We propose to approximate **D** by **B**. This reduces the required number of operations to *O*(*K*).

### Estimation of the attachment exponent

Given $\hat{A_{k}}$ and their estimated variances *v*_*k*_, if we assume that the attachment kernel is indeed *A*_*k*_ = *k* ^*α*^, it is an important task to correctly estimate the attachment exponent *α*. Jeong’s method proposes to fit *k* ^*α*+1^ to the cumulative function Π(*k*) = ∑_*j*:*j* ≤ *k*_ *A*_*j*_ in order to estimate *α*.

To take advantage of the estimated variances given by PAFit, we propose to find *α* by weighted least squares method. The variance of $\text{log }{\hat{A}}_{k}$ can be estimated by $var(\text{log }A_{k})\approx v_{k}/{\hat{A}}_{k}^{2}$. The uncertainties in $\text{log }{\hat{A}}_{k}$ can be naturally incorporated into the fitting process by using weights that are inversely proportional to variances of $\text{log }{\hat{A}}_{k}$. In particular, we minimize the following objective function with respect to *α* and *d*:

$$\sum_{k}\frac{1}{var(\log{\hat{A}}_{k})}{\left(\log{\hat{A}}_{k}-\alpha \log k-d\right)}^{2}.$$

It is important to note that the idea of using estimated variances as weights in weighted least squares methods can also be applied when estimating parameters of models that are different from the model *A*_*k*_ = *k* ^*α*^.

### Binning

For more stable estimation of the attachment kernel, we use logarithmic binning in order to let the *A*_*k*_ borrow information from the ones that are near them. In logarithmic binning, the length of the *i*-th bin is *c* times the length of the (*i* − 1)-th bin. We freely choose the number of bins *B*, then *c* is determined from *B* and the maximum degree *K*. We set *A*_*k*_ = *ω*_*i*_ for all *k* in the *i*-th bin. Choosing *B* small helps with stabilizing the estimation result at the risk of loosing fine details in the attachment kernel. For the sake of simplicity we have been using *A*_*k*_ in our equations, but the readers should keep in mind that unless stated otherwise, we always use binning, so in fact *A*_*k*_ should be replaced by *ω*_*i*_.

### Regularization

After binning is performed, another important technique to reduce the variances of the estimated result is regularization. We add the following regularization term

$$\begin{array}{c} -\lambda \frac{1}{\sum_{k=1}^{K-1}w_{k}}\sum_{k=1}^{K-1}w_{k}{\left(\log A_{k+1}+\log A_{k-1}-2\log A_{k}\right)}^{2} \end{array}$$ (18)

to the log-likelihood function *l*(**A**) (see Eq (5)), and maximize the resulted function. This penalty term penalizes the second order differentiation of log*A*_*k*_, and by doing so, it encourages linearity in log*A*_*k*_.

The weight *w*_*k*_ can be any positive number. In this paper, we set *w*_*k*_ to be proportional to the number of data points of the degree *k*: $w_{k}\propto \sum_{t=1}^{T}m_{k}(t)$. Intuitively, we want to emphasize the regularization where there is plenty of data (when *k* is small) and de-emphasize it where the data is scarce (when *k* is large).

We are still able to derive an MM algorithm for maximizing the penalized log-likelihood function. The details are given in S1 Appendix. As a property of MM algorithms, the penalized log-likelihood function is also guaranteed to increase with number of iterations.

Turning now to how to choose *λ*, the ratio of the strength of the regularization term (measured by *λ*) and the number of observed data (measured by $\sum_{t=1}^{T}\sum_{k=0}^{K}m_{k}(t)$) can be used as a heuristic, reasonable criterion for choosing *λ*. The ‘right’ amount of regularization depends on many factors: the number of data points for each degree, the number of time-steps, the shape of the true attachment kernel, etc. We found that a reasonable amount of regularization with ratio ranges from 0 to 1 often leads to satisfactory results.

## Results and Discussion

### Monte Carlo simulation

Here we compare five methods: Jeong’s method, Newman’s method, the corrected Newman’s method, PAFit without regularization (ratio = 0) and PAFit with regularization (ratio = 0.1). We examine two different bin settings: *B* = 100 and *B* = 20. And we perform comparisons in three different functional forms of the true attachment kernels *A*_*k*_. Table 2 shows the true attachment kernels we used in this experiment. For each true attachment kernel, we generate 100 networks according to the GT model. Starting from a seed network with 20 nodes, at each time-step, *m*(*t*) = 5 new edges and *n*(*t*) = 1 new node were added, until a total of 2000 nodes is reached. We compare the average relative error, defined as $\frac{1}{K}\sum_{k=1}^{K}\frac{{\left(A_{k}-{\hat{A}}_{k}\right)}^{2}}{A_{k}^{2}}$, between the true value *A*_*k*_ and ${\hat{A}}_{k}$.

**Table 2:** Summary of true attachment kernels used in the Monte Carlo simulation.

Attachment kernel	Parameter	Value
*A*_*k*_ = max(*k*, 1)^*α*^	*α*	0.5, 0.6, …, 1.5 (11 values)
*A*_*k*_ = min(100, max(*k*, 1))^*β*^	*β*	0.8, 1.0, 1.2
*A*_*k*_ = 3(log(max(*k*, 1)))^*b*^ + 1	*b*	2, 3

Summary of true attachment kernels used in the Monte Carlo simulation. There is a total of 16 different kernels.

We note some remarks about the implementation of the methods. First, due to the inherent sparse nature of Jeong’s method, even with binning, sometimes the estimated result of Jeong’s method has more 0 than those of the other methods. In such cases, to ensure fair comparison, we perform linear interpolation on the log scale to interpolate the value of those zero-value ${\hat{A}}_{k}$. Second, in Jeong’s method we choose *T*_0_ as the time when the 1500-th node is added, *T*_1_ = *T*_0_ + 1 and Δ*T* = 500. Third, convergence condition for PAFit is *ϵ* = 10^−5^.

**Fig 2:**
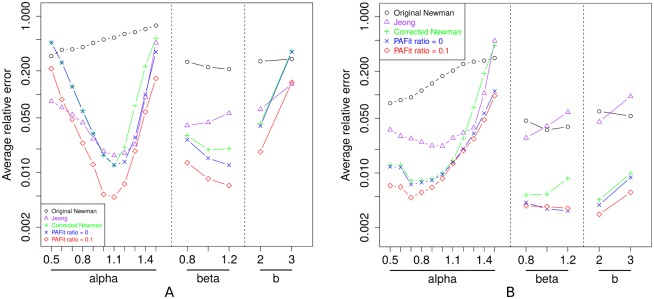
Comparison between five methods in average relative error. A: *B* = 100. B: *B* = 20. See Table 2 for the details of the true attachment kernels *A*_*k*_ used here.

The result is shown in Fig 2. Overall, PAFit with regularization outperformed all remaining methods. This suggests that a small amount of regularization is indeed needed to reduce the error in the estimated result. We also notice that binning helps reduced the error in all methods. The fewer number of bins we used, the better the estimated result was found to be.

PAFit without regularization performed better than the corrected Newman’s method. In the functional form *A*_*k*_ = *k* ^*α*^, when *α* is up to about 1.2, PAFit without regularization delivered almost the same error as that of the corrected Newman’s method, but when *α* > 1.2, PAFit without regularization was better, especially when the number of bins is 20. In the remaining two functional forms, PAFit without regularization also performed better or at least similar to the corrected Newman’s method.

When *B* = 100, except the functional form *A*_*k*_ = min(100, max(*k*, 1))^*β*^, Jeong’s method performed reasonably well in comparison with PAFit without regularization and the corrected Newman’s method. When *B* = 20, Jeong’s method became worse than those two methods. Newman’s method (uncorrected) performed the worst in almost all cases, due to its underestimating *A*_*k*_ when *k* is large.

### The Flickr social network

**Fig 3:**
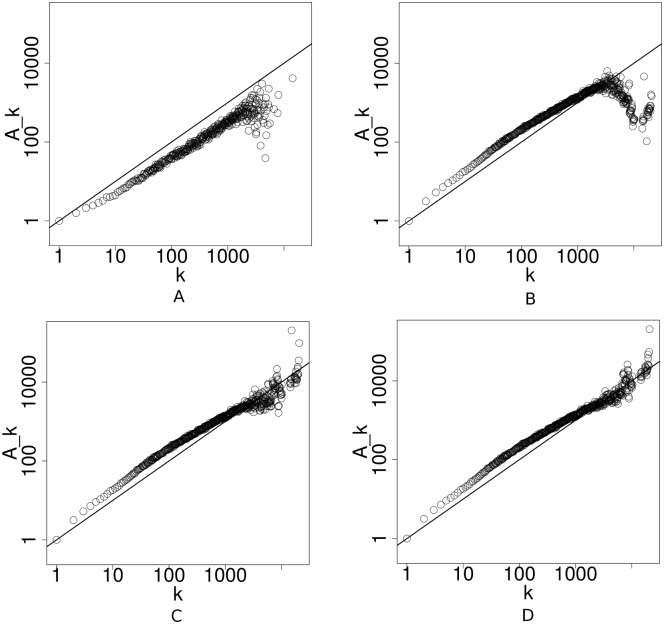
Estimation of the attachment kernel in the Flickr social network dataset. A: Jeong’s method. B: Newman’s method. C: Corrected Newman’s method. D: PAFit. The plots are on a log-log scale. The solid line corresponding to *A*_*k*_ = *k* is plotted as a visual guide.

In this section we present the results from our analysis of a publicly available Flickr social network dataset [35]. It consists of a simple directed network of friendship relations between Flickr users. The dataset contains *T* = 133 days of growth. Table 3 shows some important summary statistics of the dataset. The results are shown in Fig 3. We also implemented a quasi-Newton speed-up scheme [51] for the MM algorithms used in PAFit. The convergence condition for PAFit is *ϵ* = 10^−7^.

**Table 3:** Summary statistics for the Flickr social network dataset.

∣*V*∣	∣*E*∣	*T*	Δ∣*V*∣	Δ∣*E*∣	$\hat{\gamma }$
2302925	33140018	134	815867	16105211	2.15

Summary statistics for the Flickr social network dataset [35]. This is a directed simple network. The numbers ∣*V*∣ and ∣*E*∣ are the total number of nodes and edges in the final network, respectively. Meanwhile, *T* is the number of observed time-steps, while Δ∣*V*∣ and Δ∣*E*∣ are the increments of nodes and edges after time *t* = 0, respectively. The value $\hat{\gamma }$ is the power-law exponent of the degree distribution of the final network [6].

For Jeong’s method, we use all the available data by choosing *T*_0_ = 0, *T*_1_ = 1 and Δ*T* = 132. It found a sub-linear attachment kernel ${\hat{A}}_{k}$. The value of ${\hat{A}}_{k}$ fluctuated considerably for large value of *k*, however. Note that the domain of ${\hat{A}}_{k}$ of Jeong’s method is smaller than in other methods, since the degrees of *T*_0_-nodes are fixed at *T*_0_.

In the estimated result of Newman’s method, we once again spotted the falling off ${\hat{A}}_{k}$ when *k* is large. This phenomenon is, of course, completely eliminated in the corrected Newman’s method.

For PAFit, we performed regularization with ratio equal to 0.1. Although the estimation result of PAFit looks very similar to that of the corrected Newman’s method, the ${\hat{A}}_{k}$ in the high degree region of PAFit are less fluctuated and more compact than those of the corrected Newman’s method. It is worth noting that we spotted a clear signal of deviation from the log-linear model *A*_*k*_ = *k* ^*α*^ here.

## Conclusion

We proposed a statistically sound method, called PAFit, for estimating the attachment kernel *A*_*k*_ in temporal networks. The method is nonparametric in the sense that it does not assume any particular functional form for *A*_*k*_. In this way it is able to detect different types of functional forms. We proved that the log-likelihood function is concave under a suitable re-parametrization, and provided a Minorize-Maximization algorithm for its maximization. The proposed algorithm is shown to increase the log-likelihood function monotonically with the number of iterations. It also has the property that if it converges, it will converge to global maximum of the log-likelihood function. We also investigated binning and regularization, and showed that these two simple techniques considerably improve the quality of the estimation. We reported clear evidence for the presence of PA in the Flickr social network. We also found that the functional form of the attachment kernel differs from the classically assumed log-linear form, *A*_*k*_ = *k* ^*α*^.

In this paper, we focused on estimating the attachment kernel. Another important ingredient related to temporal network evloution is node fitness [52–55]. In the future, we would like to consider a natural generalization of the method of this paper, in which we can propose a method to estimate jointly the attachment kernel and node fitness. Another potential research direction is theoretical justifications of the superiority of PAFit over Jeong’s method and Newman’s method. We expect that PAFit and its future extensions will become useful tools for analyzing many types of temporal networks in the real-world.

## Supporting Information

S1 Appendix Derivation of the MM algorithm when the regularization term in Eq (18) is added. (PDF)
