# Supplementary material for: PAFit: A Statistical Method for Measuring Preferential Attachment in Temporal Complex Networks
Source: PLoS One. 2015 Sep 17;10(9):e0137796. doi: 10.1371/journal.pone.0137796 (PMC4574777; doi:10.1371/journal.pone.0137796)
Supplement: S1 Appendix — (PDF) [file pone.0137796.s001.pdf]

Here we derive the update for A_k when the regularization term in Eq. (18) is added. The new formula for $A_k^{(i+1)}$ is not available in closed form. This $A_k^{(i+1)}$ is the solution of a rather complicated equation. First we need to derive a minorize function of Eq. (18). The strategy is the same as what can be found in [1]. For a concave function $g(x)$, we have the following inequality that comes directly from the definition of a concave function.

$$g\left(\frac{1}{2}m+\frac{1}{4}n+\frac{1}{4}p\right) \geq \frac{1}{2}g(m)+\frac{1}{4}g(n)+\frac{1}{4}g(p).$$

By choosing $g(x) = -w_k x^2$ and

$$\begin{aligned} m &= -4\log A_k + 2\log A_k^{(i)} + \log A_{k+1}^{(i)} + \log A_{k-1}^{(i)}, \\ n &= 4\log A_{k+1} + \log A_{k-1}^{(i)} - 2\log A_k^{(i)} - 3\log A_{k+1}^{(i)}, \\ p &= 4\log A_{k-1} - 2\log A_k^{(i)} + \log A_{k+1}^{(i)} - 3\log A_{k-1}^{(i)}, \end{aligned}$$

we have

$$\begin{aligned} &-\lambda\frac{1}{\sum_k w_k}\sum_k w_k\left(\log A_{k+1}+\log A_{k-1}-2\log A_k\right)^2 \\ &\geq -\lambda\frac{1}{\sum_k w_k}\sum_k\Bigg(\frac{w_k}{2}\left(-4\log A_k + 2\log A_k^{(i)} + \log A_{k+1}^{(i)} + \log A_{k-1}^{(i)}\right)^2 + \\ &\frac{w_k}{4}\left(4\log A_{k+1} + \log A_{k-1}^{(i)} - 2\log A_k^{(i)} - 3\log A_{k+1}^{(i)}\right)^2 + \\ &\frac{w_k}{4}\left(4\log A_{k-1} - 2\log A_k^{(i)} + \log A_{k+1}^{(i)} - 3\log A_{k-1}^{(i)}\right)^2\Bigg). \end{aligned} \tag{S1}$$

One can verify that the right hand side of Eq. (S1) is a minorize function of Eq. (18). Combining this function with the minorize function of the log-likelihood (see Eq. (10)), we get a minorize function for the penalized log-likelihood function at iteration i. Let call this final minorize function Q. The whole point of deriving Eq. (S1) is that the variables A_0, A_1,..., A_K in Q are separable. This means that by using Q, at each iteration i, we can turn a $K+1$-variable maximization problem into $K+1$ problems in which each is univariate, and thus can be easily solved. Specifically, each equation $\partial Q/\partial A_k = 0$ is univariate with A_k being the sole variable. $A_k^{(i+1)}$ is then the solution of $\partial Q/\partial A_k = 0$.

References

[1] Hunter D, Lange K. A Tutorial on MM Algorithms. The American Statistician. 2004;58:30–37.
